# Programmable RNA-Guided DNA Recombination: Mechanisms, Engineering, and Applications

**DOI:** 10.3390/biomedicines14092008

**Published:** 2026-09-07

**Authors:** Ahmed S. A. Ali Agha, Dima Hattab, Athirah Bakhtiar, Arwa Omar Al Khatib, Heba Salah Abushahla, Salma Alketbi, Amal Akour

**Affiliations:** 1 Pharmacological and Diagnostic Research Centre, Faculty of Pharmacy, Al-Ahliyya Amman University, Amman 19328, Jordan; asat3u@gmail.com; 2 School of Pharmacy, Department of Pharmaceutical Sciences, The University of Jordan, Amman 11942, Jordan; dym9220264@ju.edu.jo (D.H.); hbh9220302@ju.edu.jo (H.S.A.); 3 School of Pharmacy, Monash University Malaysia, Jalan Lagoon Selatan, Bandar Sunway 47500, Selangor, Malaysia; 4 Faculty of Pharmacy, Al-Ahliyya Amman University, Amman 19328, Jordan; a.alkhatib@ammanu.edu.jo; 5 Department of Pharmacology and Therapeutics, College of Medicine and Health Sciences, United Arab Emirates University, Al Ain 15551, United Arab Emirates; 202132236@uaeu.ac.ae; 6 Department of Biopharmaceutics and Clinical Pharmacy, School of Pharmacy, The University of Jordan, Amman 11942, Jordan

**Keywords:** seekRNA, bridge RNA, RNA-guided recombination, bridge recombinase, IS110, ISCro4, programmable genome engineering, site-specific recombination, large-payload genome editing, double-strand break-independent genome editing, CRISPR-associated transposases

## Abstract

The emergence of seekRNA- and bridgeRNA-guided recombination has introduced a distinct paradigm in genome engineering by coupling programmable RNA-directed DNA recognition with recombinase-mediated insertion, excision, inversion, and genomic rearrangement without canonical double-strand breaks. Since their discovery in 2024, these systems have progressed rapidly from bacterial mobile genetic elements and mechanistic characterization to structural elucidation and programmable genome engineering in human cells. However, these advances remain distributed across foundational and rapidly emerging studies, creating a need for an integrated molecular perspective on their mechanisms, technological development, and position within contemporary genome engineering. This review synthesizes the molecular architecture, RNA-guided recognition, strand-exchange mechanisms, programmability, and engineering of seekRNA and bridgeRNA systems, with particular emphasis on complementary human-cell advances involving ISCro4 and engineered IS621. Whereas ISCro4 systems have enabled multikilobase DNA insertion, genomic excision, and near-megabase inversion, the enIS621–tebRNA platform has enabled scarless kilobase-scale integration across multiple human cell types, including proof-of-concept functional CD19 chimeric antigen receptor and factor IX gene insertion. The review further integrates recent genome-scale bacterial rewriting and Targetable Recombinase Assisted DNA Exchange (TRADE)-mediated DNA replacement, while benchmarking RNA-guided recombination against conventional site-specific recombination, clustered regularly interspaced short palindromic repeats (CRISPR)-based editing, Programmable Addition via Site-specific Targeting Elements (PASTE), CRISPR-associated transposases, and emerging large-payload genome-writing strategies, including kilobase-scale nickase-targeting (KNIT) editing, Prime Assembly, engineered R2 retrotransposons, and TransCRISTI. This comparative framework highlights a broader transition from programmable sequence modification toward direct engineering of genomic architecture, while identifying recognition-site constraints, mismatch-tolerant recombination, unintended recombination products, delivery, and genome-wide specificity as key translational barriers. By integrating foundational mechanisms with recent mammalian engineering, genome-scale bacterial rewriting, large-payload technologies, and emerging computational design strategies, this review provides a contemporary framework for defining the distinctive capabilities, current limitations, and future development of programmable RNA-guided DNA recombination.

## 1. Introduction

The ability to precisely manipulate genomic DNA represents one of the most transformative advances in modern molecular biology. Since the elucidation of the DNA double helix, genome engineering has evolved from restriction enzyme-mediated DNA manipulation to highly programmable molecular platforms capable of targeted gene disruption, correction, insertion, and chromosomal rearrangement across diverse biological systems [1]. These advances have profoundly expanded our understanding of gene function while accelerating discoveries in functional genomics, synthetic biology, biotechnology, and precision medicine.

The first generation of programmable genome-editing technologies emerged through engineered nucleases, including zinc-finger nucleases (ZFNs) and transcription activator-like effector nucleases (TALENs), which established the feasibility of sequence-specific genomic modification [2]. The subsequent development of clustered regularly interspaced short palindromic repeats (CRISPR)/CRISPR-associated (Cas) systems fundamentally transformed genome engineering by coupling RNA-guided target recognition with programmable DNA cleavage [3,4,5]. This innovation dramatically improved editing efficiency, versatility, and accessibility. Collectively, these advances established programmable genome editing as a central platform in modern molecular biology.

**Figure 1:**
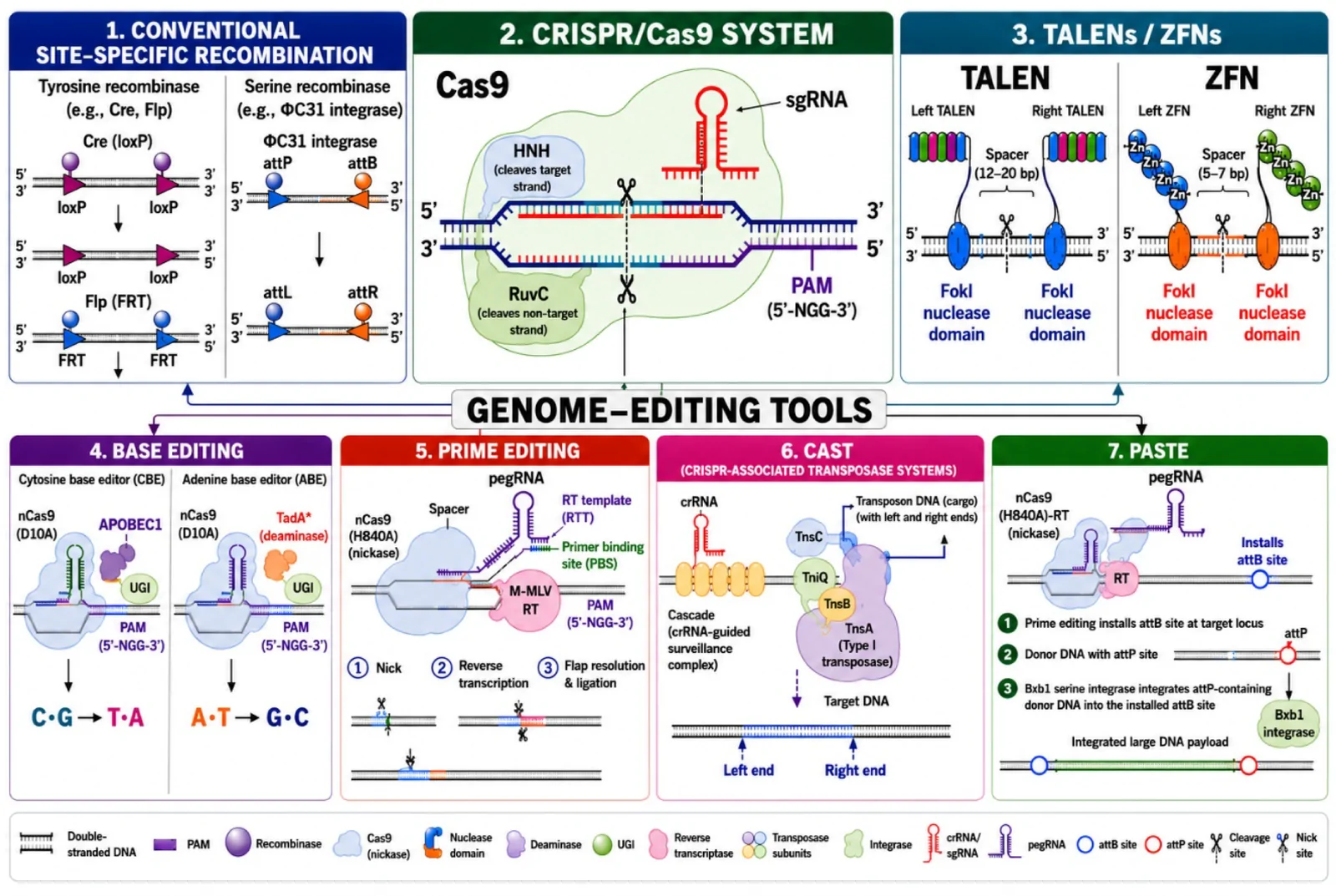
Overview of major genome-editing technologies. Schematic comparison of the molecular architectures and mechanisms of representative genome-editing platforms. (1) Conventional site-specific recombination is mediated by tyrosine recombinases (Cre/loxP and Flp/FRT) or serine integrases (e.g., ΦC31), which catalyze DNA recombination between specific recognition sites. (2) The CRISPR/Cas9 system employs an sgRNA to direct PAM-dependent target recognition and Cas9-mediated DNA double-strand cleavage. (3) TALENs and zinc-finger nucleases (ZFNs) utilize engineered DNA-binding proteins fused to the FokI nuclease, requiring dimerization for targeted DNA cleavage. (4) Base editors combine Cas9 nickase with nucleotide deaminases to mediate programmable C•G→T•A or A•T→G•C base conversion without DNA double-strand breaks. (5) Prime editors fuse Cas9(H840A) nickase with a reverse transcriptase and a prime-editing guide RNA (pegRNA) to introduce precise substitutions and small insertions or deletions without donor DNA or double-strand cleavage. (6) CRISPR-associated transposase (CAST) systems couple RNA-guided target recognition with transposase-mediated insertion of DNA cargo into defined genomic loci. (7) PASTE combines prime editing with Bxb1 serine integrase to enable programmable insertion of large DNA payloads through attB/attP site-specific recombination. Schematics are not drawn to scale.

Despite their remarkable success, most current genome-editing technologies remain fundamentally dependent on DNA double-strand break (DSB) formation and endogenous DNA repair pathways. Repair through non-homologous end joining (NHEJ) or homology-directed repair (HDR) frequently produces variable editing outcomes, including unintended insertions, deletions, and chromosomal rearrangements, while limiting the efficient integration of large DNA cargoes [6,7,8,9]. Consequently, considerable effort has been devoted to developing DSB-independent editing technologies. These include base editors, prime editors, Programmable Addition via Site-specific Targeting Elements (PASTE), and CRISPR-associated transposases (CAST), all of which seek to improve editing precision while reducing genotoxicity [10,11]. Nevertheless, these platforms remain constrained by limited cargo capacity, delivery complexity, incomplete editing efficiency, or multi-component molecular architectures (Figure 1).

Importantly, programmable RNA-guided recombination should be viewed as an expansion rather than a replacement of existing genome-engineering technologies. Durrant et al. first publicly described a bispecific bridge RNA encoded by IS110-family elements and demonstrated that its target- and donor-binding regions could be independently reprogrammed to mediate DNA insertion, excision, and inversion [12]. Independently, Siddiquee et al. showed that non-coding-region-derived seekRNAs guide target selection by IS1111- and IS110-family elements and that coordinated redesign of the seekRNA and donor flank can redirect insertion to alternative sites [13]. Unlike cleavage-dependent genome-editing technologies, these compact systems couple programmable RNA-directed DNA recognition to recombinase-mediated strand exchange without canonical DNA double-strand breaks or extensive dependence on endogenous DNA-repair pathways. Together, these independent studies established programmable RNA-guided DNA recombination as a distinct framework for repair-independent genome engineering [12,13].

The field has evolved rapidly since these foundational discoveries. Perry et al. identified and engineered ISCro4 for programmable human-genome rearrangement, enabling multikilobase DNA insertion, precise chromosomal excision, and scar-free inversions approaching megabase scale [14]. Pelea et al. subsequently provided an independent demonstration of ISCro4-mediated editing in human cells and used cryo-electron microscopy to define structural determinants of enhanced activity and guide-RNA engineering [15]. Guo et al. later engineered the initially characterized IS621 recombinase and its cognate bRNA to generate enIS621–tebRNA, which supported scarless integration of kilobase-scale circular DNA donors across multiple human cell types and enabled proof-of-concept functional CD19-CAR and *F9 integration* [16].

In parallel, compact type V-K CAST systems, laboratory-evolved CAST variants, and Prime Assembly have expanded the repertoire of DSB-independent large-payload genome-engineering technologies through distinct integration chemistries [17,18,19]. Collectively, these advances illustrate a broader transition from cleavage-dependent sequence editing toward programmable engineering of genomic architecture.

Given these rapid advances, an updated molecular synthesis of the field is timely. This review aims to critically synthesize the molecular architecture, recombination mechanisms, engineering advances, and emerging applications of seekRNA- and bridgeRNA-guided recombination while positioning these systems within the rapidly evolving landscape of genome-engineering technologies. Particular emphasis is placed on their mechanistic distinctions, programmability, and critical comparison with established and emerging genome-editing platforms.

## 2. Mechanistic Overview of RNA-Guided Recombination Systems (seekRNA and bridgeRNA)

RNA-guided processes play fundamental roles in diverse biological functions, including translation, gene regulation, RNA interference, and adaptive immunity. In bacteria and archaea, autonomous mobile genetic elements (MGEs) serve as major drivers of genome evolution by mobilizing insertion sequences (IS) and transposons that mediate DNA integration, excision, and horizontal gene transfer, thereby contributing to adaptive traits such as antibiotic resistance [20,21]. Recent discoveries indicate that a subset of these MGEs has evolved programmable RNA-guided recombinases, establishing a mechanistic link between classical transposition and programmable genome engineering.

Two IS families, IS110 and IS1111, have attracted particular attention because of their highly selective integration-site preferences and their use of non-coding RNAs to direct recombination [12,13]. Durrant et al. characterized the IS110-family bridgeRNA system using IS621 [12]. These bridgeRNAs typically contain two internal loops: a target-binding loop that recognizes the genomic target and a donor-binding loop that recognizes the IS donor DNA. Each loop contains guide segments that pair with both strands of its corresponding DNA substrate, and the two loops can be independently reprogrammed, allowing modular control of target and donor recognition [12].

Independently, Siddiquee et al. characterized seekRNA-mediated target selection across IS1111- and IS110-family elements [13]. These seekRNAs occur as short species and longer precursor forms, both of which contain complementary segments matching the top and bottom strands of the target DNA. Their co-purification with the cognate transposase supported formation of an active ribonucleoprotein complex, and coordinated modification of the seekRNA recognition segments and donor-flanking sequence redirected insertion to alternative target sites [13].

Both systems employ a compact DEDD recombinase containing an N-terminal RuvC-like catalytic domain and an NCR-derived guide RNA. The NCR is generally located upstream of tnp in IS110-type elements and downstream in IS1111-type elements, producing opposite guide-RNA transcriptional orientations [12,13,22].

**Figure 2:**
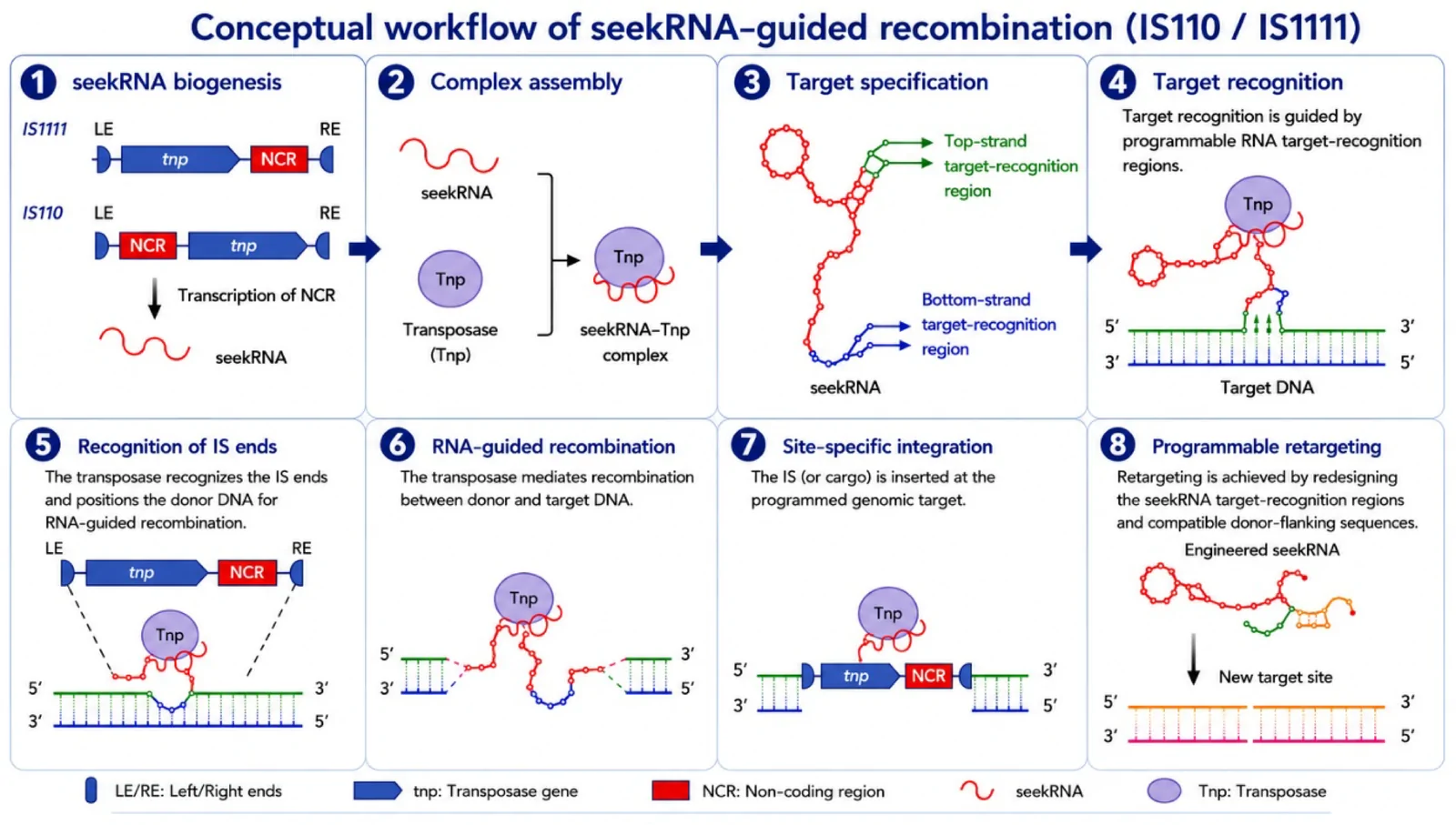
Conceptual workflow of seekRNA-guided recombination in IS110/IS1111 systems. Following transcription of the non-coding region (NCR), seekRNA associates with the transposase (Tnp) to form an active ribonucleoprotein complex. Programmable target-recognition regions within seekRNA determine genomic target specificity, enabling recognition of matching DNA sequences. The transposase recognizes the insertion-sequence (IS) ends, brings donor and target DNA into proximity, catalyzes RNA-guided recombination, and mediates site-specific integration. Reprogramming the target-recognition regions enables redirection of recombination to alternative genomic loci. This schematic summarizes the currently proposed mechanism based on available experimental evidence.

Following transposon excision and circularization, a junction-associated promoter drives transcription of the functional guide RNA from the circular intermediate [12,13]. Structural analysis of the IS621 system further showed that bridgeRNA scaffolds a tetrameric synaptic complex containing donor and target DNA and that recombination proceeds through sequential strand exchange, with top-strand exchange preceding bottom-strand exchange [23]. The principal molecular events underlying seekRNA-guided recombination are summarized in Figure 2.

**Figure 3:**
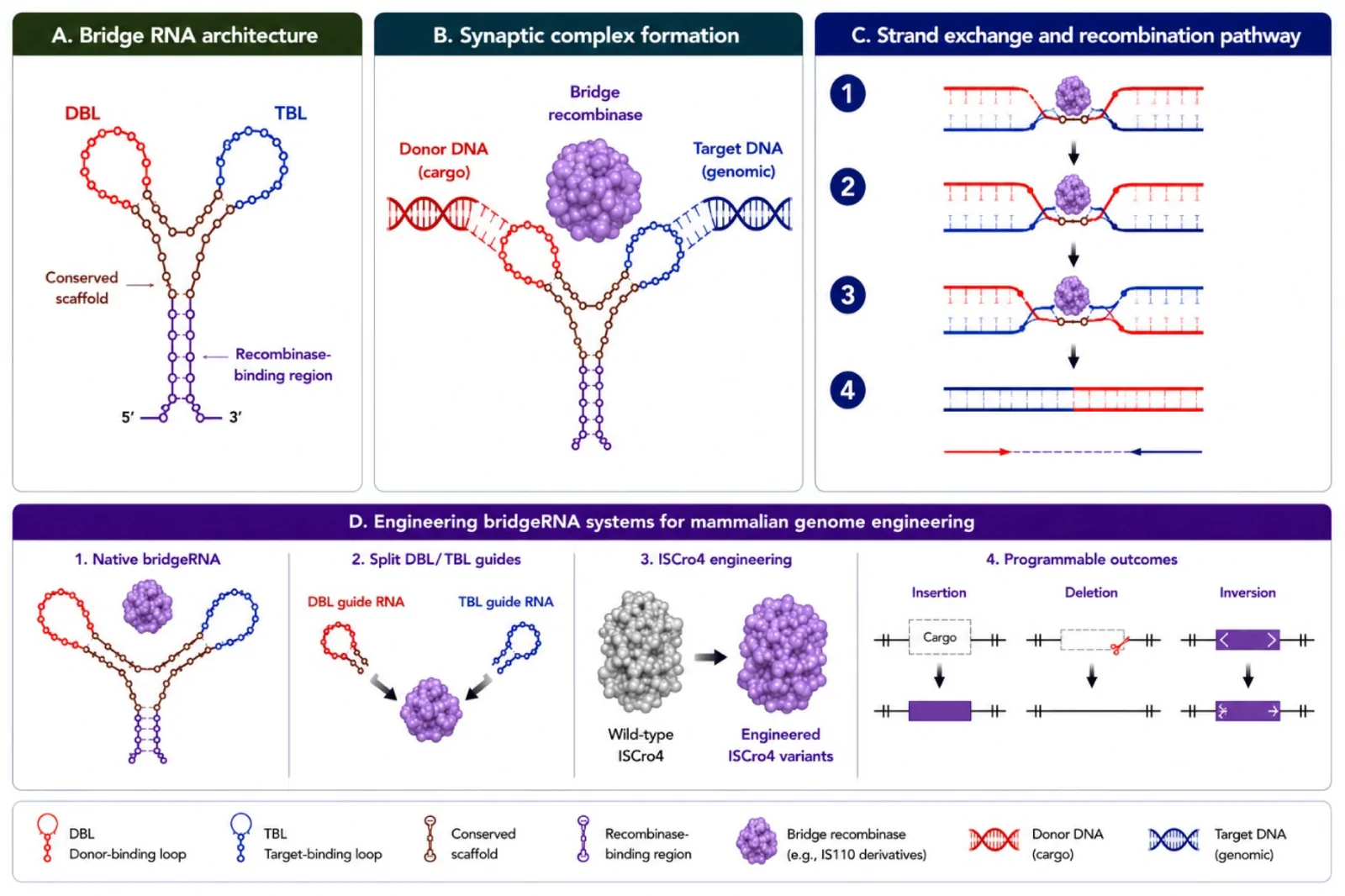
BridgeRNA-guided recombination: architecture, synaptic complex, and engineering. (**A**) Generalized architecture of bridgeRNA showing the donor-binding loop (DBL), target-binding loop (TBL), conserved scaffold, and recombinase-binding region. (**B**) The bridgeRNA simultaneously base-pairs with donor and target DNA to recruit the bridge recombinase and assemble the synaptic complex. (**C**) Proposed recombination pathway involving sequential strand exchange through a junction intermediate, culminating in site-specific integration of donor DNA. (**D**) Engineering strategies for mammalian genome editing, including native bridgeRNA, split DBL/TBL guide RNAs, engineered ISCro4 recombinases, and representative programmable outcomes including targeted insertion, deletion, and inversion. The schematic summarizes current mechanistic understanding based on available structural and functional studies.

Cryo-electron microscopy further revealed that recombination occurs within a tetrameric synaptic complex in which bridgeRNA functions as the central molecular scaffold coordinating simultaneous donor DNA recognition, target DNA recognition, and recombinase assembly. The recombinase shields transient DNA intermediates throughout sequential strand exchange, providing a structural explanation for the absence of canonical DNA double-strand breaks and the minimal indel formation characteristic of bridgeRNA-mediated recombination [15]. These structural features and their translation into engineered mammalian genome-editing systems are summarized in Figure 3.

Translation of bridgeRNA-guided recombination into human cells was initially demonstrated by Perry et al., who identified ISCro4 as a highly active platform for programmable genome rearrangement [14]. Engineered ISCro4 supported scar-free multikilobase DNA insertion, chromosomal excision, and inversions approaching megabase scale while enabling systematic evaluation of activity, sequence discrimination, and unintended recombination products [14].

Pelea et al. independently identified ISCro4 as a highly active human-cell bridge recombinase and provided structural insights into the basis of its enhanced activity [15]. Additional protein–RNA and protein–DNA interactions stabilized the synaptic complex, whereas the linker connecting the donor-binding and target-binding loops contributed minimally to assembly. This observation enabled separation of bridgeRNA into independent donor- and target-binding guide RNAs, a split-guide architecture that improved recombination efficiency while preserving programmable recognition [15].

A complementary strategy was subsequently applied to IS621, combining protein substitutions that enhance association with bRNA and DNA with a two-part, loop-exchanged bRNA architecture to generate the enIS621–tebRNA platform for large-fragment insertion in human cells [16].

The IS110 and IS1111 systems exemplify a remarkably compact RNA-guided recombination architecture composed of a single recombinase (approximately 300–456 amino acids) and a single programmable non-coding RNA (Table 1).

Unlike nuclease-dependent genome-editing platforms, seekRNA and bridgeRNA systems catalyze targeted DNA insertion, excision, inversion, and rearrangement without introducing canonical DNA double-strand breaks while maintaining a compact molecular architecture that may facilitate vector delivery [12,13].

Building upon this native mechanism, engineering of the ISCro4 bridge recombinase successfully translated RNA-guided recombination into programmable genome editing in human cells, enabling scar-free multi-kilobase DNA insertion, chromosomal excision, and inversions approaching megabase scale [14,15].

In *E. coli*, reprogrammed IS621 bRNAs directed 51.6–94.0% of detected insertions to their intended sites, although off-target integrations occurred most frequently at sequences containing one or two mismatches, and rare deletions and inversions were detected between insertion sites [12]. Subsequent human-cell studies identified a similarly nuanced specificity profile, including mismatch-tolerant genomic recombination and unintended donor–donor or target–target products, indicating that catalytic activity, product purity, and sequence discrimination remain coupled engineering challenges [14,15].

Collectively, structural, biochemical, and engineering studies establish seekRNA and bridgeRNA as a fundamentally distinct class of programmable recombination systems. Their repair-independent mechanism, compact molecular architecture, and successful translation into mammalian genome editing position RNA-guided recombination as a promising next-generation platform for genome engineering, synthetic biology, and therapeutic applications.

## 3. Programmability and Engineering of RNA-Guided Recombination Systems

Beyond their unique recombination mechanism, a defining feature of seekRNA and bridgeRNA is their programmability. By redesigning discrete sequence elements within the guide RNA, both systems can be redirected to new genomic loci without modifying the recombinase itself, enabling highly flexible and programmable genome engineering.

Durrant et al. demonstrated that the target- and donor-binding loops of bridgeRNA can be reprogrammed independently [12]. Redesign of the target-binding loop redirected IS621 recombination to alternative genomic sites while abolishing activity at the native target, whereas coordinated redesign of the donor-binding loop and donor sequence enabled programmable recognition of alternative DNA cargoes. Genome-wide analysis showed that more than half of detected insertions occurred at the intended site, whereas most off-target integrations mapped to sequences containing one or two mismatches relative to the programmed target. The system also supported insertion of multikilobase DNA donors as well as programmable excision and inversion [12].

Independently, Siddiquee et al. demonstrated that both long and short seekRNA species could be reprogrammed by modifying their target-complementary regions together with the corresponding flanking sequences in the IS donor, enabling insertion into newly specified genomic sites [13]. Collectively, these studies established RNA-encoded sequence recognition as a general principle of programmable recombination: genomic retargeting can be achieved principally through guide-RNA and donor-sequence redesign rather than recombinase re-engineering [12,13]. This modularity subsequently provided a foundation for systematic development of bridge recombinases in mammalian cells [14,15].

Recent work has expanded bridge recombination beyond the initial bacterial proof-of-concept systems. Na et al. described ISPpu10 as a structure-gated bridge recombinase that drives large-scale genomic plasticity in *Pseudomonas putida* [24]. In this system, bRNA–DNA complementarity defines the genomic address, whereas a 5′ target-DNA hairpin acts as an efficiency gate. ISPpu10 enabled cross-species chromosomal integration, mobilization of a 22.9-kb plasmid cargo, and genomic capture of split donor segments in trans, thereby extending bridge recombination to sequence-and-structure-gated targeting [24].

Patel et al. subsequently demonstrated genome-scale IS621-mediated editing in *E. coli*, including insertions approaching 142 kb, excisions of approximately 50 kb, and inversions up to 2.3 Mb [25]. The system also supported integration across 11 bacterial species and within enriched human gut microbial communities. In the same study, Targetable Recombinase Assisted DNA Exchange (TRADE) used two bRNAs to mediate homologous-recombination-independent replacement of genomic segments, including an approximately 50.4-kb colibactin biosynthetic gene cluster [25].

Guo et al. then reported engineering of the original IS621 platform for human cells [16]. The enIS621–tebRNA system mediated scarless integration of kilobase-scale circular DNA donors across multiple cell lines, retained measurable activity with a 10-kb donor, and enabled proof-of-concept functional CD19-CAR and *F9 integration*. However, genome-wide mapping identified candidate off-target integrations, emphasizing the need for further specificity optimization and validation in primary cells and in vivo models [16].

**Figure 4:**
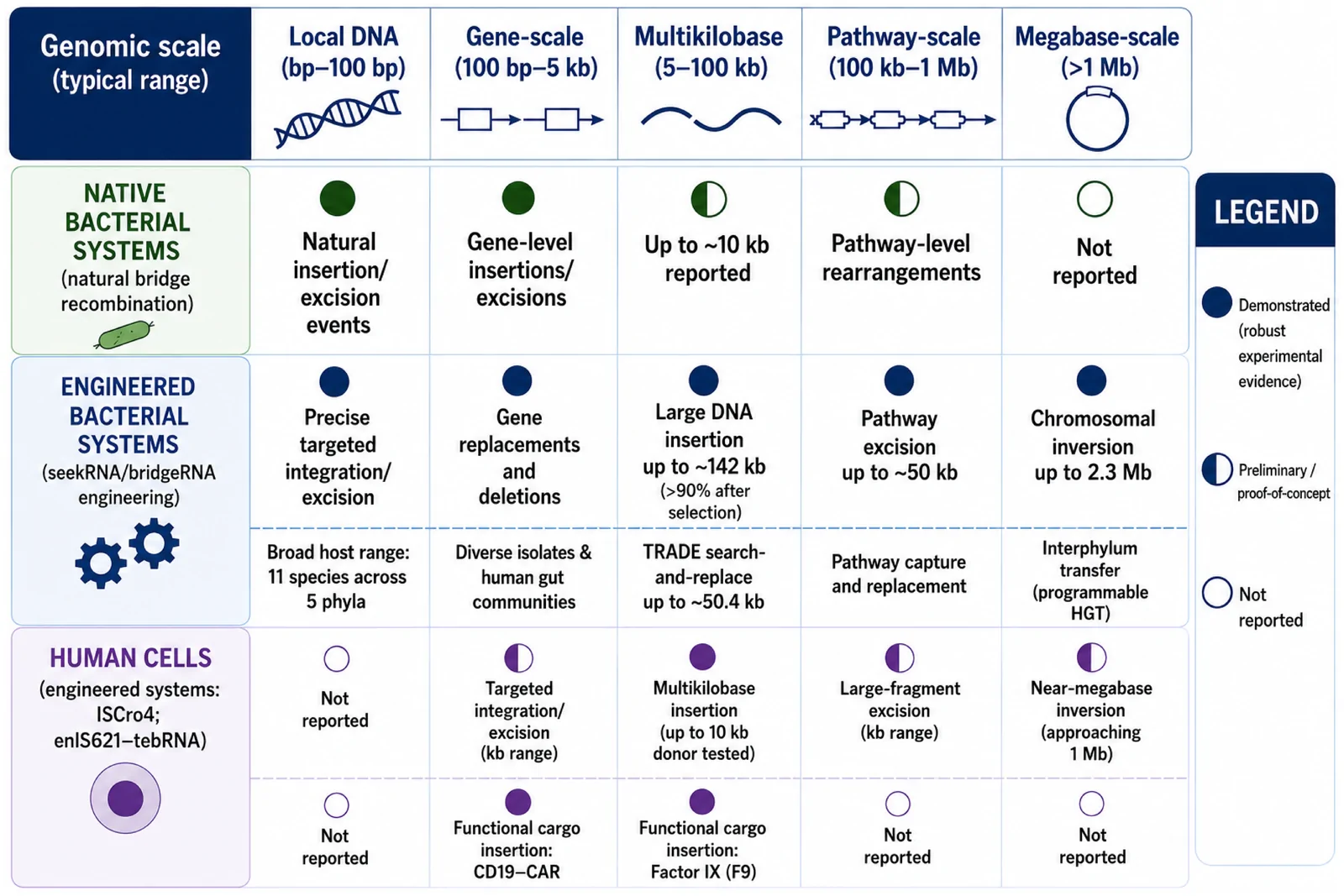
Genomic scale and experimental maturity of RNA-guided recombination across bacterial and human systems. The matrix summarizes the demonstrated capabilities of native bacterial, engineered bacterial, and human-cell systems across increasing scales of genome manipulation, from local DNA editing to megabase-scale rearrangement. Filled, half-filled, and open circles indicate robust experimental evidence, preliminary/proof-of-concept evidence, and capabilities not yet demonstrated, respectively. Genomic-scale ranges are approximate and reflect representative reported capabilities.

Together, these advances illustrate the expanding scale and experimental maturity of RNA-guided recombination across bacterial and human systems, as summarized in Figure 4.

## 4. Comparative Analysis with Established Genome-Editing Technologies

To place RNA-guided recombination within the broader landscape of genome engineering, it is essential to compare these systems with established and emerging genome-editing platforms. Although all are designed to enable programmable manipulation of genomic DNA, they differ fundamentally in their molecular mechanisms, reliance on DNA cleavage and endogenous repair pathways, targeting flexibility, engineering complexity, and editing capabilities (Table 2).

### 4.1. Conventional Site-Specific Recombination

Conventional site-specific recombination (SSR) is mediated principally by tyrosine recombinases, including Cre and Flp, and large serine recombinases (LSRs), such as Bxb1 and ΦC31 [27,30,46,47,48]. These enzymes recognize defined pairs of DNA sites and catalyze repair-independent strand exchange through covalent protein–DNA intermediates. Tyrosine recombinases exchange strands sequentially through a Holliday-junction intermediate, whereas LSRs cleave all four strands and exchange them by subunit rotation. Recognition-site identity and orientation determine whether the outcome is integration, excision, or inversion, and LSRs can support directionally controlled integration of large DNA cargos without exposed DSBs [30].

Despite their dependence on predetermined recognition sites, conventional site-specific recombination systems have been substantially expanded through recognition-site engineering, enzyme discovery, and protein optimization. Zhang et al. used model-guided engineering of the Bxb1 attP sequence to tune recombination kinetics across approximately four orders of magnitude, with optimized variants achieving up to a tenfold increase in initial reaction rate relative to the wild-type site [49]. Durrant et al. subsequently expanded the large-serine-recombinase repertoire through systematic microbial mining, identifying more than 60 recombinases experimentally characterized in human cells; selected enzymes achieved 40–75% integration of cargoes larger than 7 kb and up to sevenfold greater activity than Bxb1 [27].

Further diversification was achieved through protein and recognition-site engineering. Mukhametzyanova et al. inserted zinc-finger DNA-binding domains into Cre-type recombinases to generate target-dependent enzymes requiring recognition of both the recombinase site and an adjacent zinc-finger-binding sequence [50]. Cautereels et al. identified 16 orthogonal LoxPsym variants suitable for multiplexed recombination after systematic screening of 1192 pairwise combinations [51]. Hew et al. then used phage-assisted continuous evolution to generate hyperactive ΦC31 and Bxb1 integrases, with evolved Bxb1 variants achieving up to 80% targeted integration and 56.2% integration of a 15.7-kb therapeutic cargo [52].

More recent studies have partly overcome the requirement for a pre-installed cognate attachment site. Fanton et al. engineered Dn29 variants capable of direct integration at an endogenous human pseudosite, achieving up to 53% integration efficiency and 97% genome-wide specificity with cargoes up to 12 kb [28].

Fauser et al. subsequently developed the modular integrase framework MINT to retarget Bxb1 to endogenous AAVS1 and *TRAC* loci, where optimized constructs supported efficient large-cargo integration in K562 cells and primary human T cells [29]. These findings demonstrate that conventional recombinases are increasingly engineerable rather than intrinsically fixed.

### 4.2. Programmable DNA Nucleases and CRISPR-Derived Editors

#### 4.2.1. DSB-Based Programmable Nucleases: ZFNs, TALENs, and CRISPR–Cas Systems

Programmable DNA nucleases marked the transition from conventional site-specific recombination to customizable genome engineering by coupling engineered DNA-recognition modules with catalytic nuclease activity. Zinc-finger nucleases (ZFNs), transcription activator-like effector nucleases (TALENs), and CRISPR–Cas systems represent successive generations of programmable nuclease technologies that enable targeted DNA cleavage followed by endogenous repair through non-homologous end joining (NHEJ) or homology-directed repair (HDR) [53]. Although these platforms employ distinct DNA-recognition strategies, they all fundamentally rely on the programmable induction of DNA double-strand breaks (DSBs) to achieve genome modification.

ZFNs were the first programmable DNA nucleases developed for targeted genome editing. They comprise engineered zinc-finger DNA-binding domains, each recognizing a specific DNA triplet, fused to the FokI endonuclease domain [54]. Binding of two ZFN monomers to adjacent target sites promotes FokI dimerization and generates a site-specific DSB that is subsequently repaired through endogenous NHEJ or HDR pathways [55,56,57,58]. This modular architecture established ZFNs as an early platform for customizable genome modification in mammalian cells. In a representative study, Xu et al. designed ZFNs targeting the porcine *ApoE* gene and demonstrated their sequence-specific activity in HEK293T cells, providing further evidence that engineered ZFNs can mediate targeted genome modification in mammalian systems [59]. Despite their versatility and potential for high specificity, the widespread application of ZFNs has been constrained by the complexity of protein engineering, context-dependent interactions among adjacent zinc-finger modules, and the potential for off-target cleavage and cytotoxicity [60].

TALENs were subsequently developed to overcome several design limitations associated with ZFNs. These nucleases employ transcription activator-like effector (TALE) repeats that recognize individual nucleotides through a comparatively straightforward one-repeat-to-one-nucleotide recognition code [61]. Similar to ZFNs, TALENs use FokI-mediated dimerization to generate targeted DSBs that are repaired through endogenous NHEJ or HDR pathways [55,56,57,58]. Their modular DNA-recognition code simplified target design and improved targeting flexibility. Shankar et al. demonstrated the therapeutic potential of TALENs by disrupting the *E7* oncogene of human papillomavirus type 16 (HPV-16), resulting in marked suppression of *E7* protein expression in cervical cancer cells [62]. Nevertheless, TALENs still require the construction of a distinct protein pair for each genomic target and retain the inherent limitations of DSB-dependent genome editing, including the risks of off-target cleavage, undesired repair outcomes, and chromosomal instability [60].

The emergence of CRISPR–Cas systems represented a further major advance by replacing protein-based target recognition with RNA-guided DNA recognition, thereby substantially increasing the programmability and scalability of genome editing. The canonical CRISPR–Cas9 editing system comprises three principal components: (1) a single-guide RNA (sgRNA), generated by combining the targeting functions of CRISPR RNA (crRNA) and trans-activating crRNA (tracrRNA), which directs Cas9 to a complementary DNA sequence; (2) the Cas9 endonuclease, which cleaves the target and non-target DNA strands to generate a predominantly blunt-ended DSB approximately three base pairs upstream of the protospacer-adjacent motif (PAM); and (3) the PAM itself, which is required for target recognition and cleavage. As with ZFNs and TALENs, the resulting DSB is repaired by endogenous NHEJ or HDR pathways, enabling targeted gene disruption or sequence modification.

Early applications established the versatility of CRISPR–Cas9 in human disease modeling. Paquet et al. used CRISPR–Cas9 to introduce precise heterozygous and homozygous mutations into human induced pluripotent stem cells, generating Alzheimer’s disease models that reproduced genotype-dependent neuronal phenotypes [63]. Subsequent assessments of therapeutic genome editing highlighted persistent limitations, including low HDR efficiency, unintended repair products, delivery constraints, off-target cleavage, and potential immune responses to CRISPR-associated components [32]. Despite these challenges, continued optimization of CRISPR-based platforms enabled their progression into clinical practice. In 2023, the UK Medicines and Healthcare products Regulatory Agency authorized Casgevy—an ex vivo CRISPR–Cas9 therapy targeting the erythroid-specific enhancer of *BCL11A*—for eligible patients with sickle cell disease or transfusion-dependent β-thalassemia. This authorization represented the first regulatory approval of a CRISPR-based therapy.

The CRISPR toolbox was subsequently expanded through the characterization of Cas homologs with distinct targeting and cleavage properties. Cas12a, formerly known as Cpf1, is an RNA-guided DNA endonuclease that recognizes PAM sequences different from those used by Cas9 and generates staggered DSBs at PAM-distal positions, thereby providing alternative targeting and cleavage patterns [64]. In contrast, Cas13 proteins are RNA-guided RNA-targeting nucleases capable of programmable transcript recognition and degradation rather than DNA cleavage [65,66].

Although Cas12 and Cas13 broaden the targeting scope of CRISPR technologies, their activity can also extend beyond the intended substrate. Ai et al. reported that Cas13 effectors exhibit variable collateral and off-target RNA-cleavage profiles across eukaryotic cell types, emphasizing the importance of effector selection, cellular context, and application-specific optimization when these systems are used experimentally or therapeutically [67]. Collectively, ZFNs, TALENs, and CRISPR–Cas systems established the central principle of programmable genome targeting; however, their reliance on nuclease-mediated cleavage and endogenous repair also motivated the development of editing technologies designed to introduce defined sequence changes without conventional DSB formation.

#### 4.2.2. Base Editors (CBEs and ABEs)

Base editors and prime editors represent second-generation CRISPR/Cas technologies that employ Cas9 nickase (nCas9) to introduce a single-strand DNA nick instead of DSBs, thereby minimizing DNA damage and improving repair fidelity. These systems expand the precision and safety of genome editing compared with conventional nuclease-based CRISPR platforms [33,34].

Base editors enable targeted single-nucleotide substitutions without relying on DSBs, donor DNA templates, or HDR. Two principal classes, cytosine base editors (CBEs) and adenine base editors (ABEs), mediate C•G to T•A and A•T to G•C transitions, respectively [33,34]. Both utilize a catalytically inactive or nickase Cas9 (dCas9/nCas9) fused to a single-stranded DNA deaminase domain. Upon binding the target DNA, the Cas9-sgRNA complex forms an R-loop, exposing the non-complementary strand. In CBEs, cytosines within the editing window are deaminated to uracils (read as thymines), while in ABEs, adenosines are deaminated to inosines (read as guanines). Glycosylase inhibitors prevent the excision of uracil or hypoxanthine bases, avoiding error-prone repair. The nickase activity of Cas9 directs repair of the opposite DNA strand, leading to fixation of the intended base [33,34].

Continuous optimization of base editors has improved efficiency, targeting scope, and product purity. Engineered Cas9 variants with expanded PAM compatibility and Cas12a homologs have broadened the accessible genomic space, increasing the proportion of pathogenic ClinVar mutations correctable from 26% to 95% [68,69]. Moreover, modifications to deaminase domains influence the editing window and conversion efficiency [70,71]. Critical considerations for effective base editor implementation include: (1) selecting an appropriate editor class, (2) identifying PAM sequence constraints, (3) evaluating local sequence context, (4) optimizing editing window parameters, (5) minimizing off-target deamination, and (6) validating editing outcomes for product purity [4].

#### 4.2.3. Prime Editors (PEs)

Prime editors build upon the single-strand nicking concept to enable the precise insertion, deletion, or substitution of defined DNA sequences at virtually any genomic locus [35]. Unlike base editors, which are limited to transition mutations, prime editors employ a reverse-transcription mechanism that supports a far broader spectrum of edits. The system consists of two core components: (1) a fusion protein (PE2) comprising Cas9 nickase linked to an engineered reverse transcriptase, and (2) a prime editing guide RNA (pegRNA) that specifies both the target site and the desired edit sequence [4].

Upon binding to the genomic target, the PE2 complex nicks the PAM-containing strand, generating a 3′ end that hybridizes with the primer-binding sequence on the pegRNA. The reverse transcriptase then extends this 3′ terminus, synthesizing a DNA segment encoding the intended edit. Cellular repair pathways subsequently remove the displaced 5′ flap containing the unedited sequence and incorporate the newly synthesized strand, forming a heteroduplex that resolves into the edited product. To increase conversion efficiency, a secondary nick on the non-edited DNA strand, guided by an sgRNA, facilitates replacement of the remaining wild-type DNA [4].

Prime editing offers exceptional versatility, enabling all 12 possible base substitutions, targeted insertions and deletions, and sequence modification more than 30 bp from the pegRNA-induced nick site [35]. Importantly, prime editors exhibit substantially reduced off-target effects compared with DSB-based nucleases, producing fewer indels and minimizing genomic instability [35]. Their combination of precision, editing range, and minimal DNA cleavage establishes prime editors as one of the most promising next-generation tools for therapeutic genome engineering.

Beyond insertion and rearrangement, the recent development of dual-bRNA TRADE extends bridge recombination to HR-independent, pathway-scale search-and-replace editing in bacteria, providing a mechanistically distinct counterpart to prime-editing-derived strategies for programmable DNA replacement [25].

### 4.3. CRISPR-Associated Integration Techniques

Beyond base and prime editors, CRISPR-directed systems now support targeted kilobase-scale integration through several distinct biochemical routes. PASTE installs an attachment site by prime editing before serine-integrase-mediated recombination, CAST recruits a transposase to a CRISPR-defined genomic locus, and CRISPR kilobase-scale nickase-targeting (KNIT) editing recruits homology-armed donor DNA to a Cas9-nickase-generated single-strand break [10,17,18,39]. Although all three approaches avoid a programmed genomic double-strand break, they differ substantially in donor architecture and repair requirements: PASTE and CAST use integrase- or transposase-catalysed insertion independently of canonical HDR, whereas KNIT promotes localized capture of a homology-armed donor at a single nick. These distinctions are important when comparing component burden, insertion chemistry and genomic by-products.

#### 4.3.1. Programmable Addition via Site-Specific Targeting Elements

PASTE (Programmable Addition via Site-specific Targeting Elements) combines a Cas9 nickase fused to a reverse transcriptase (RT) with a site-specific serine integrase such as Bxb1 [10]. The process proceeds into two coordinated stages. First, RT-mediated prime editing installs a short attB “landing pad” sequence at the target locus. Second, the integrase catalyzes recombination between this attB site and a circular donor DNA containing the complementary attP sequence. Early PASTE versions achieved approximately 15% integration efficiency for 1-kb transgenes in human cells. Through systematic protein engineering, optimizing fusion architectures and identifying more than 25,000 integrase homologs, the enhanced PASTEv3 variant now supports transgene integrations up to 36 kb at 10–20% efficiency, with markedly reduced off-target activity compared to DSB-dependent methods such as HITI [10].

#### 4.3.2. CRISPR-Associated Transposase (CAST)

In contrast, CAST systems couple CRISPR-guided target recognition with transposase-driven DNA integration [38]. Initial translation of bacterial CAST systems to human cells was limited by low activity and the challenge of assembling multicomponent transposition machinery in a eukaryotic environment.

Liu et al. demonstrated that compact type V-K CAST systems, which use a single Cas12k targeting effector rather than a multiprotein Cascade complex, can mediate programmable integration of therapeutically relevant DNA cargo in multiple human cell types [17]. Witte et al. subsequently reported evoCAST, generated by phage-assisted continuous evolution of type I-F CAST components [18].

The evoCAST achieved approximately 10–30% integration of kilobase-scale cargoes across multiple human genomic loci and accommodated DNA fragments of at least 10 kb [18].

Prime Assembly provides a further, mechanistically distinct benchmark by combining paired prime editing with overlapping linear DNA donors to enable multikilobase insertion without a recombinase or programmed DSB [19].

These developments provide complementary benchmarks for RNA-guided recombinases by demonstrating alternative routes to programmable large-payload integration with distinct requirements for targeting components, donor architecture, and integration chemistry.

Taken together, PASTE and CAST extend the CRISPR framework toward kilobase-scale, site-specific genomic integration while mitigating genotoxic side effects characteristic of DSB-based systems. Nevertheless, both platforms remain mechanistically complex, relying on multi-component protein assemblies and, in many cases, engineered attachment or integration sites. In contrast, the recently discovered seekRNA and bridgeRNA recombinase systems achieve programmable, multi-kilobase DNA insertions using a single small recombinase and a single RNA guide, without requiring canonical DNA cleavage or pre-installed recombinase recognition sites, although sequence constraints on donor and target recognition remain. This streamlined architecture positions RNA-guided recombination as a distinct emerging genome-engineering paradigm that combines programmable RNA-directed target recognition with autonomous, repair-independent DNA recombination.

#### 4.3.3. CRISPR Kilobase-Scale Nickase-Targeting Editing

More recently, Gao et al. developed CRISPR kilobase-scale nickase-targeting (KNIT) editing as a DSB-free strategy for programmable kilobase-scale DNA integration [39]. Unlike PASTE and CAST, which rely on integrase- or transposase-catalysed insertion, KNIT enhances otherwise inefficient HDR at a single DNA nick by recruiting donor DNA directly to the targeted locus. In KNIT editor 1 (KE1), monomeric streptavidin fused to SpCas9(D10A) nickase recruits a biotinylated, homology-armed dsDNA donor. Integration required donor homology and the HDR factors RAD51 and BRCA2 and exhibited cell-cycle regulation, supporting a proximity-enhanced single-nick HDR mechanism [39]. KNIT editor 2 (KE2) further employs a 24×GCN4 SunTag/scFv–streptavidin architecture to recruit multiple donor molecules and enhance integration efficiency.

Across multiple genomic loci and cell types, KNIT supported insertion of 0.7-kb to >10-kb DNA fragments, with efficiencies reaching approximately 89% in selected experiments, while substantially reducing indels, chromosomal translocations, and genome-wide off-target insertions relative to Cas9-nuclease-mediated knock-in [39]. The system also enabled repeated and multilocus integration and restored wild-type *LIPA* expression through targeted insertion at either the AAVS1 safe-harbour or native *LIPA* locus. In primary human T cells, KNIT improved viability and proliferative yield relative to Cas9–dsDNA editing and reduced markers associated with cGAS–STING activation and apoptosis. KE2 further achieved non-viral CAR integration at the *TRAC* locus at 32.9 ± 8.2% efficiency (up to 40.6%), with the resulting CAR-T cells exhibiting antitumour activity in vitro and in a xenograft model [39].

KNIT therefore represents a mechanistically distinct DSB-free integration strategy that requires neither an integrase nor a transposase. However, unlike bridgeRNA-guided recombination, KNIT remains dependent on Cas9/PAM recognition, donor homology arms, and endogenous HDR, potentially reducing its efficiency in non-dividing cells [39]. Its current implementation also requires engineered donor recruitment, while the scalability of PCR-generated biotinylated dsDNA and the immunogenicity of synthetic components remain to be established. Conversely, bridge recombinases catalyse direct donor–target strand exchange without conventional homology arms or host HDR and can remain active in cell-cycle-arrested human cells, although their primary-cell validation and therapeutic development remain less mature. Accordingly, KNIT and bridgeRNA systems represent complementary routes to DSB-free large-payload genome engineering based on proximity-enhanced nick-triggered HDR and direct RNA-programmed recombination, respectively.

### 4.4. Emerging DSB-Free and Hybrid Genome-Writing Strategies

Persistent limitations of conventional CRISPR nucleases, particularly DSB-associated indels, chromosomal rearrangements, and dependence on endogenous repair, have stimulated the development of mechanistically diverse genome-writing strategies that avoid or reduce reliance on programmed DSBs. Representative examples include twin prime editing (twinPE) and Prime Assembly, engineered R2 retrotransposons, and the CRISPR–transposase fusion TransCRISTI [19,37,40,41,42,43,44,45]. These platforms employ distinct donor formats and integration chemistries—including prime-editing-generated DNA flaps, RNA-templated reverse transcription, and transposase-mediated insertion—and therefore provide complementary benchmarks for evaluating the compact, direct strand-exchange mechanism of bridgeRNA-guided recombination.

#### 4.4.1. Twin Prime Editing

Twin prime editing (twinPE) extends conventional prime editing to larger and more complex genomic alterations by using two pegRNAs to generate complementary 3′ DNA flaps at distinct genomic positions [36,37]. Annealing and resolution of these programmed flaps enable targeted sequence replacement and excision without introducing a programmed DSB. The twinPE system achieved sequence replacement efficiencies approaching 80% using paired pegRNAs; when combined with the Bxb1 site-specific serine recombinase, it further enabled targeted integration of gene-sized DNA payloads up to 5.6 kb and genomic inversions spanning 40 kb [36,37]. By avoiding nuclease-generated DSBs, twinPE reduces the indels and chromosomal rearrangements associated with conventional cleavage-dependent editing. However, its paired-pegRNA architecture increases design complexity, editing efficiency remains sequence- and locus-dependent, and large-payload integration and large genomic inversion in the original implementation require an auxiliary recombinase. These limitations have motivated subsequent prime-editing-derived strategies, including Prime Assembly, that directly couple programmable 3′-flap formation to multikilobase donor incorporation without requiring a site-specific recombinase.

#### 4.4.2. Prime Assembly

Prime Assembly extends twinPE-derived paired-flap editing to direct multikilobase donor integration. In the approach reported by Liu et al., paired pegRNAs generate complementary genomic 3′ flaps that anneal to short overlapping sequences engineered at the ends of linear double-stranded DNA donors [19]. One or multiple overlapping donor fragments can consequently undergo Gibson-like assembly directly at the target locus, enabling insertions ranging from approximately 0.1 to 11.3 kb. Inhibition of DNA-PK-dependent end joining increased both insertion efficiency and product precision, while activity in non-cycling cells supported independence from canonical HDR. The method requires neither a recombinase nor an exogenous DNA-dependent DNA polymerase but retains the delivery and design requirements of a prime editor, two pegRNAs, and one or more appropriately terminated linear DNA donors [19].

A separate Prime Assembly (PA) framework reported by Jung et al. generates complementary 3′ flaps on both genomic and donor DNA using single-flap (SF-PA), nick-enhanced single-flap (SFn-PA), or dual-flap (DF-PA) configurations [40]. These formats accommodated DNA donors ranging from 1.0 to 6.5 kb, with DF-PA achieving up to 57.8% replacement using a 2.9-kb donor in HEK293T cells; long-read sequencing showed intact donor incorporation in 77.5–93.7% of donor-integrated reads across the three configurations. DF-PA additionally coupled donor insertion to deletion of endogenous regions spanning 100 kb or 1 Mb, albeit at lower efficiencies, while PA supported non-viral CAR integration in primary human T cells at up to 28.1%, producing functional CAR-T cells with antitumour activity [40]. Mechanistic perturbation implicated RAD51, BRCA1 and BRCA2, supporting a homology-directed process despite the absence of programmed DSBs. PA therefore enables multikilobase insertion and large-segment replacement without an integrase, but remains limited by prime-editor and multi-guide complexity, large-donor delivery, dsDNA-associated cytotoxicity and imperfect products such as flap damage or adjacent genomic deletions [40].

#### 4.4.3. Engineered R2 Retrotransposons

Engineered R2 non-LTR retrotransposons provide an RNA-templated route to targeted gene addition through target-primed reverse transcription. Zhang et al. developed precise RNA-mediated insertion of transgenes (PRINT), which uses an R2-protein-encoding mRNA and a transgene-template RNA to direct insertion at the multicopy 28S rDNA locus [41]. PRINT accommodated templates up to 4 kb, and more than 50% of cultured human cells acquired several 2-kb insertions, more than half of which were full length [41].

Chen et al. subsequently developed en-R2Tg through combined protein and donor-RNA engineering [42]. The system achieved greater than 20% integration in human cells, supported all-RNA delivery, retained greater than 10% integration with approximately 2.5-kb donors, and showed high on-target specificity at the 3′ junction. Nevertheless, incomplete 5′ junctions and low-frequency on-target indels remained detectable [42].

Fell et al. extended R2 targeting beyond the native rDNA locus through STITCHR, which combines SpCas9(H840A) nickase with a truncated, naturally reprogrammable R2 protein [43]. The platform enabled scarless edits ranging from single-nucleotide changes to insertions up to 12.7 kb at multiple endogenous loci and remained active in dividing and non-dividing cells [43].

Edmonds et al. later used structural and biochemical insights to minimize the R2Tg donor, chemically stabilize its 5′ end, and optimize lipid-nanoparticle delivery [44].

The resulting compact all-RNA system achieved high integration efficiencies in human cell lines, although efficiency declined with increasing cargo size and truncated products remained the predominant imperfect outcome [44].

Collectively, engineered R2 platforms offer the distinctive advantages of RNA donor delivery and direct RNA-templated genomic synthesis. Native all-RNA systems remain primarily directed to multicopy rDNA, whereas STITCHR extends targeting to other genomic loci at the cost of a larger Cas9–R2 fusion, PAM-compatible targeting, and homology-programmed RNA donors [41,42,43,44].

#### 4.4.4. TransCRISTI: CRISPR–piggyBac Hybrid Integration

TransCRISTI combines CRISPR-directed genomic cleavage with piggyBac-mediated donor excision and tethering. The system fuses *Streptococcus pyogenes* Cas9 nuclease to an excision-competent but integration-deficient piggyBac transposase (PBdm; R372A/K375A), which releases and tethers an ITR-flanked donor. Following sgRNA-directed Cas9 cleavage, the donor is positioned near the genomic DSB and incorporated through homology-independent cellular repair; accordingly, the donor requires neither homology arms nor sgRNA-binding sites for linearization [45]. In HEK293T cells, TransCRISTI achieved site-directed integration efficiencies of 4.13% and 3.69% at the AAVS1 and *PML* loci, respectively. Among selected AAVS1-derived isogenic clones, targeted insertion was detected in 72% of TransCRISTI clones compared with 44% using HITI [45]. The system also showed fewer random and detected off-target integrations than HITI under the experimental conditions. However, because genomic integration remains dependent on Cas9-generated DSBs and subsequent cellular repair, TransCRISTI is mechanistically distinct from DSB-independent genome-writing approaches and from the direct strand-exchange mechanism of bridgeRNA-guided recombination. Its validation also remains limited to HEK293T cells and a small number of genomic loci, while the large Cas9–PB fusion may complicate delivery [45].

#### 4.4.5. Comparative Positioning of bridgeRNA-Guided Recombination

The bridgeRNA-guided recombination occupies a distinct editing regime and should be viewed as complementary rather than universally superior to existing technologies. Relative to seekRNA, bridgeRNA has undergone more extensive structural characterization and human-cell engineering, whereas mammalian seekRNA activity remains unresolved [12,13,14,15,16,23]. Compared with conventional site-specific recombination, redesign of the bridgeRNA target- and donor-binding loops enables locus and donor retargeting without evolving a new recombinase or pre-installing a cognate attachment site; conventional recombinases nevertheless remain more mature, and engineered integrases such as Dn29 and MINT can achieve efficient large-cargo integration at selected endogenous loci [28,29]. Bridge recombination also avoids programmed DSBs and repair-mediated editing associated with ZFNs, TALENs and CRISPR–Cas nucleases, although these platforms remain substantially more validated [32,53,60].

Conversely, base and prime editors remain preferable for nucleotide substitutions and short precise edits, for which bridge recombinases have not demonstrated comparable resolution or design maturity [33,34,35], whereas twinPE enables DSB-independent sequence replacement and excision using paired pegRNAs but requires coupling to a site-specific serine recombinase for gene-sized DNA integration and large genomic inversion [37].

Among large-payload approaches, bridge recombinases combine a compact RNA-programmable architecture with direct donor–target strand exchange and require neither a PAM, pre-installed attB/attP site, conventional homology arms nor reverse transcription [12,15]. PASTE supports larger payloads but requires sequential prime-editing and integrase steps [10], whereas CAST relies on CRISPR-associated transposition machinery [17,18]. KNIT achieves efficient DSB-free knock-in but remains dependent on Cas9 nickase, PAM recognition, donor homology and endogenous HDR [39], while Prime Assembly requires prime-editor machinery, multiple guides and specifically configured donors [19,40]. Engineered R2 systems can support RNA-only donor delivery, although native implementations remain largely restricted to rDNA and broader STITCHR targeting requires a Cas9–R2 fusion [41,42,43,44]. TransCRISTI likewise remains dependent on Cas9-mediated DSB formation and cellular repair [45].

These architectural advantages do not establish bridgeRNA as the most efficient or safest platform. Human-cell performance remains locus- and cargo-dependent: enIS621–tebRNA achieved 27.75% integration with kilobase-scale cargo but only 2.53% with a 10-kb donor [16]. PAM independence also does not imply unrestricted targeting, because recombination remains constrained by the core dinucleotide, guide–DNA pairing geometry and local sequence context [12,15]. Mismatch-tolerant off-target recombination and unintended donor–donor or target–target products remain important specificity and product-purity limitations [14,15], while enIS621–tebRNA also showed candidate off-target integrations and markedly reduced efficiency with a 10-kb donor [16].

Moreover, IS621 exhibits a pronounced insertion–excision bias. In vitro, excision occurred at 0.03% compared with 63% insertion, corresponding to an approximately 2000-fold difference, and bRNA modifications that enhanced insertion concomitantly reduced excision [26]. The cryo-EM attributed this asymmetry partly to substrate geometry: the U-shaped donor–target DNA configuration favours top-strand exchange during insertion, whereas the X-shaped excision complex renders this step less efficient. Directionality is nevertheless tunable because handshake-guide (HSG)–DNA base pairing modulates the favourability of top-strand exchange, providing an RNA-level parameter for engineering insertion versus excision outcomes [26].

Thus, the current comparative value of bridgeRNA-guided recombination lies in its compact RNA-programmable architecture, direct repair-independent strand exchange, and capacity for diverse structural genome edits, rather than demonstrated superiority in efficiency, payload capacity, specificity, or translational readiness.

### 4.5. Ethical and Regulatory Outlook

The emergence of RNA-guided DNA recombination technologies, such as seekRNA and bridgeRNA, introduces transformative possibilities for genome engineering while simultaneously demanding rigorous ethical and regulatory scrutiny. These systems offer distinctive capabilities for programmable genome manipulation, but their novel mechanisms raise important questions regarding safety, governance, and equitable implementation.

A central concern involves the possibility for unintended genomic alterations [72]. Despite their high target specificity, even minor off-target rearrangements could have significant biological consequences, particularly in complex or therapeutically relevant genomes. As with earlier CRISPR-based interventions, comprehensive preclinical validation and transparent oversight are essential to ensure reproducibility and long-term genomic stability.

Another ethically sensitive area concerns germline modification. While somatic editing offers immense therapeutic potential, heritable alterations invoke intergenerational ethical dilemmas and societal debate [73]. Adherence to international frameworks, such as those established by the WHO, UNESCO, and national bioethics councils, remains crucial to prevent misuse and to maintain public confidence in responsible innovation.

Equitable access and biosecurity also warrant attention. The technical sophistication required for RNA-guided recombinase systems may amplify disparities between well-resourced and developing regions [74]. Ensuring equitable access to these technologies requires global cooperation, open-science initiatives, and cost-effective delivery platforms. At the same time, the inherent programmability of RNA-guided recombinases heightens biosecurity risks, underscoring the need for robust monitoring frameworks to prevent unauthorized genetic manipulation or dual-use exploitation.

As these RNA-guided recombination tools converge with CRISPR-derived platforms such as PASTE and CAST, integrated ethical governance will be critical. Policymakers, researchers, and ethicists must collaborate to develop unified standards encompassing safety, transparency, and societal accountability. Responsible stewardship of these technologies should extend beyond laboratory compliance to address global equity, intellectual property, and environmental impact.

Ultimately, seekRNA and bridgeRNA exemplify the next frontier of genome rewriting, capable of profound therapeutic benefit yet demanding a commensurate level of ethical foresight. Their continued development should proceed under a governance model grounded in transparency, inclusivity, and shared responsibility, ensuring that scientific progress remains aligned with societal values and public trust [75,76]. With continued innovation and strong ethical governance, seekRNA and bridgeRNA could inaugurate a new paradigm of repair-independent programmable genome editing, a frontier poised to redefine both research and clinical genomics.

## 5. Future Directions

Computational design is emerging along two complementary fronts: direct generation of bridge recombinases and prediction of bRNA performance. EDEN-BR was fine-tuned on more than six million putative bridge-recombinase-containing genomic regions to generate recombinase sequences from bRNA prompts [77]. Pilot cell-free testing identified two active candidates among 49 designs, which retained predicted ISCro4-like structures despite sharing only 22.4% and 34.5% sequence identity with ISCro4. These findings provide preliminary evidence that foundation models can explore functional recombinase sequence space beyond natural homolog mining, although the editing activity, target specificity, and safety of the generated recombinases have not yet been validated in cells [77].

Complementing direct recombinase generation, predictive computational models could improve bRNA design. Current evidence indicates that recombination efficiency and specificity vary substantially among bRNAs and genomic contexts and remain difficult to predict from sequence information alone. Patel et al. therefore proposed coupling large-scale bRNA libraries with machine-learning approaches to determine how target-sequence context, local transcriptional activity, chromosomal topology, and proximity to the origin of replication influence recombination outcomes [25]. Such datasets could support predictive models of bRNA efficiency and specificity and ultimately reduce the present dependence on empirical guide screening. In the longer term, integrating sequence-based learning with structural information on bRNA–DNA–recombinase complexes may enable rational optimization of guide architecture, orthogonality, and multiplex compatibility.

Beyond computational design, the major experimental priority is to determine whether the mechanistic advantages of RNA-guided recombination can be translated into robust and predictable editing across therapeutically relevant mammalian cell types and genomic contexts. Future studies should systematically define donor- and target-sequence constraints, genome-wide specificity, mismatch tolerance, unintended donor–donor and target–target recombination, and the consequences of large-scale genomic rearrangements. Parallel development of compact delivery strategies and orthogonal bRNA–recombinase pairs could facilitate multiplexed editing while reducing cross-reactivity between independently programmed recombination events. Ultimately, rigorous comparison with established DSB-independent platforms in clinically relevant models will be required to determine whether the compact architecture and large-payload capabilities of bridge recombinases translate into meaningful advantages in editing efficiency, product purity, delivery, and genomic safety.

## 6. Conclusions

Programmable seekRNA- and bridgeRNA-guided recombination represents a rapidly evolving genome-engineering paradigm that couples RNA-defined targeting with direct recombinase-mediated manipulation of genomic architecture. Since their discovery in 2024, these systems have progressed from mechanistic and structural characterization to molecular engineering, genome-scale bacterial rewriting, TRADE-mediated replacement, and multikilobase insertion, large excision, and near-megabase inversion in human cells. This review integrates these rapidly emerging developments into a unified molecular and technological framework encompassing biological origins, mechanism, programmability, structural basis, engineering, and emerging applications.

Comparison with conventional site-specific recombination and emerging genome-writing platforms, including PASTE, CAST/evoCAST, KNIT, Prime Assembly, engineered R2 retrotransposons, and TransCRISTI, positions RNA-guided recombination within the broader transition from sequence editing toward programmable engineering of genomic architecture. Its distinctive value lies in expanding the mechanistic and structural scale of programmable genome engineering through compact RNA-programmable recognition, repair-independent strand exchange, and versatile large-scale DNA manipulation. However, recognition-site constraints, mismatch tolerance, unintended recombination products, delivery, and genome-wide specificity remain major barriers. Further integration of structural, computational, delivery, and genome-wide safety approaches will be essential to determine whether these systems can progress from experimental tools to reliable biotechnology and therapeutic platforms.

## Figures and Tables

**Table 1:** Examples of IS*110* and IS*1111* family members producing seekRNA and bridgeRNA.

IS Family	IS Member	Transposase Size (aa)	seekRNA Length (nt)		Donor DNA	The Target Matches	The Insertion Points of the Target Site	The Order of the Matches	Ref.
			The Short Band	The Long Band					
IS*1111*	ISEc11	339	82	154	ISEc11	Top 5′GTGAAAATACTGT3′ Bottom 3′CACTTTTATGACA5′	TG*AA AC TT	The top strand match is 5′ to the bottom strand match	[13]
	ISPa11	N/A	96	172	ISPa11	Top 5′GTAGGGCGaATAACCGC3′ Bottom 3′CATCCCGCtTATTGGCG5′	GG*CG CC CG		[13]
	ISKpn4	N/A	82	151	ISKpn4	Top 5′CGGCTTGAACGAATTGTTAG3′ Bottom 3′GCCGAACTTGCTTAACAATC5′	AA*CG TT GC		[13]
	ISPst6	N/A	86	148	ISPst6	Top 5′CGATTTGAACGCATTGTTAG3′ Bottom 3′GCTAAACTTGCGTAACAATC5′	AA*CG TT GC		[13]
	ISPa25	N/A	82	N/A	ISPa25	Top 5′GACCTTGAGCGACTTGTTAG3′ Bottom 3′CTGGAACTCGCTGAACAATC5′	AG*CG TC GC		[13]
IS*110*	ISEc21	358aa	74	156	ISEc2	Top 5′CACAGCGATCAGGGaAG3′ Bottom 3′GAGACGCTAGTCCCtTC5′	AT*CA TA GT	The top strand match is 3′ to the bottom strand match	[13]
	IS621	IS621	The bridgeRNA (177nt)		IS621	Top 5′ATCAGGCCTACAGA3′ Bottom 3′TAGTCCGGATGTCT5′	GC*CT CG GA		[12]

Note: Purple-highlighted bases indicate regions of the top (T) DNA target strand that are complementary to the cognate guide RNA (seekRNA or bridgeRNA), whereas blue-highlighted bases indicate regions of the bottom (B) DNA target strand that are complementary to the cognate guide RNA. *: The insertion points of the target site.

**Table 2:** Comparative characteristics of established and emerging programmable genome-engineering platforms.

Platform	Mechanism	DSB/Repair	Scope/Scale	Key Strength	Key Limitation
seekRNA [13]	RNA-guided recombination	No DSB; HDR/NHEJ-independent	Integration; mainly bacterial	Compact; PAM-independent	Early-stage; mammalian activity unresolved
BridgeRNA [12,14,15,16,23,26]	bRNA-guided recombination	No DSB; HDR/NHEJ-independent	Insertion, excision, inversion; multi-kb–near-Mb	Compact donor + target RNA programmability	Sequence/directionality constraints; unintended products
Conventional SSR [27,28,29,30]	Site-specific recombination	No DSB; HDR/NHEJ-independent	Insertion, excision, inversion; multi-kb	Mature; efficient	Fixed recognition sites; protein retargeting
CRISPR–Cas9 [3,4,31,32]	RNA-guided cleavage	DSB; NHEJ/HDR	Knockout, correction, insertion	Established; readily retargeted	Genotoxicity; repair variability; PAM/off-targets
Base editors [33,34]	Cas-guided deamination	No DSB; HDR-independent	Base substitutions	Precise point editing	Limited chemistry; bystanders; PAM
Prime editors [35,36]	Cas9 nickase–RT	No DSB; no canonical HDR	Substitutions; small indels	Versatile precise rewriting	Design complexity; limited cargo
twinPE [37]	Paired 3′-flap prime editing	No DSB; no canonical HDR *	Replacement/excision; >5-kb insertion, 40-kb inversion *	Programmable sequence replacement	Paired-guide complexity; recombinase for large edits *
PASTE [10]	Prime editing + integrase	No DSB; HDR-independent	Insertion ≤36 kb	Large-cargo integration	Multicomponent; attB/attP required
CAST [17,18,38]	CRISPR-guided transposition	No targeted DSB; HDR-independent	Insertion ≥10 kb	RNA-guided large-cargo insertion	Multicomponent machinery
KNIT [39]	Cas9-nickase donor recruitment	No DSB; HDR-dependent	Knock-in >10 kb	Efficient DSB-free integration	PAM; homology/recruitment dependence
Prime Assembly [19,40]	Prime-editing flap assembly	No DSB; repair implementation-dependent	Insertion ≤11.3 kb; segment replacement	Integrase-free multikilobase insertion	Multi-guide/donor complexity
Engineered R2 [41,42,43,44]	Target-primed reverse transcription	No programmed DSB; intrinsic cleavage	RNA-templated insertion ≤12.7 kb	RNA-donor/all-RNA delivery	rDNA or Cas9–R2/PAM constraints; incomplete products
TransCRISTI [45]	Cas9 + piggyBac tethering	DSB; repair-dependent	Targeted insertion	Homology-arm-free integration	DSB-dependent; limited validation

* Large twinPE integrations/inversions require an auxiliary recombinase; DSB: DNA double-strand break; HDR: homology-directed repair; NHEJ: non-homologous end joining; PAM: protospacer-adjacent motif; CRISPR: clustered regularly interspaced short palindromic repeats; PASTE: Programmable Addition via Site-specific Targeting Elements; CAST: CRISPR-associated transposases; KNIT: kilobase-scale nickase-targeting.

## Data Availability

No new data were created or analyzed in this study. Data sharing is not applicable to this article.

## Abbreviations

Abbreviation	Definition
AAVS1	Adeno-associated virus integration site 1
ABE	Adenine base editor
attB	Bacterial attachment site
attP	Phage attachment site
bRNA	Bridge RNA
CAR	Chimeric antigen receptor
CAR-T	Chimeric antigen receptor T cell
Cas	CRISPR-associated protein
CAST	CRISPR-associated transposase
CBE	Cytosine base editor
CD19	Cluster of differentiation 19
cGAS–STING	Cyclic GMP–AMP synthase–stimulator of interferon genes
CRISPR	Clustered regularly interspaced short palindromic repeats
crRNA	CRISPR RNA
Cryo-EM	Cryo-electron microscopy
dCas9	Catalytically inactive Cas9
DBL	Donor-binding loop
DEDD	Aspartate–glutamate–aspartate–aspartate catalytic motif
DF-PA	Dual-flap Prime Assembly
DNA	Deoxyribonucleic acid
DNA-PK	DNA-dependent protein kinase
DSB	DNA double-strand break
dsDNA	Double-stranded DNA
EDEN	Environmentally derived evolutionary network
EDEN-BR	EDEN bridge-recombinase model
en-R2Tg	Engineered R2Tg
enIS621	Engineered IS621 recombinase
evoCAST	Evolved CRISPR-associated transposase
F9	Coagulation factor IX
FokI	*Flavobacterium okeanokoites* restriction endonuclease I
HDR	Homology-directed repair
HITI	Homology-independent targeted integration
HR	Homologous recombination
HSG	Handshake guide
IS	Insertion sequence
ITR	Inverted terminal repeat
KE1	KNIT editor 1
KE2	KNIT editor 2
KNIT	CRISPR kilobase-scale nickase-targeting editing
LSR	Large serine recombinase
LTR	Long terminal repeat
MGE	Mobile genetic element
MINT	Modular integrase system
mRNA	Messenger RNA
nCas9	Cas9 nickase
NCR	Non-coding region
NHEJ	Non-homologous end joining
PA	Prime Assembly
PAM	Protospacer-adjacent motif
PASTE	Programmable Addition via Site-specific Targeting Elements
PBdm	Excision-competent, integration-deficient piggyBac transposase
PCR	Polymerase chain reaction
PE	Prime editor/prime editing
PE2	Prime editor 2
pegRNA	Prime-editing guide RNA
*PML*	Promyelocytic leukemia
PRINT	Precise RNA-mediated insertion of transgenes
rDNA	Ribosomal DNA
R2Tg	R2 retrotransposon from *Taeniopygia guttata*
RNA	Ribonucleic acid
RT	Reverse transcriptase
scFv	Single-chain variable fragment
seekRNA	Seeker RNA
SF-PA	Single-flap Prime Assembly
SFn-PA	Nick-enhanced single-flap Prime Assembly
sgRNA	Single-guide RNA
SpCas9	*Streptococcus pyogenes* Cas9
SSR	Site-specific recombination
STITCHR	Site-specific target-primed insertion through targeted CRISPR homing of retroelements
TALE	Transcription activator-like effector
TALEN	Transcription activator-like effector nuclease
TBL	Target-binding loop
tebRNA	Two-part and exchange design variant of bridge RNA
Tnp	Transposase
tracrRNA	Trans-activating CRISPR RNA
*TRAC*	T-cell receptor alpha constant
TRADE	Targetable Recombinase Assisted DNA Exchange
TransCRISTI	Transposase-CRISPR mediated targeted integration
twinPE	Twin prime editing
UNESCO	United Nations Educational, Scientific and Cultural Organization
WHO	World Health Organization
ZFN	Zinc-finger nuclease

## References

[1] WareJoncas Z., Campbell J.M., Martínez-Gálvez G., Gendron W.A.C., Barry M.A., Harris P.C., Sussman C.R., Ekker S.C. (2018). Precision gene editing technology and applications in nephrology. Nat. Rev. Nephrol..

[2] Cornu T.I., Mussolino C., Cathomen T. (2017). Refining strategies to translate genome editing to the clinic. Nat. Med..

[3] Cong L., Ran F.A., Cox D., Lin S., Barretto R., Habib N., Hsu P.D., Wu X., Jiang W., Marraffini L.A. (2013). Multiplex genome engineering using CRISPR/Cas systems. Science.

[4] Anzalone A.V., Koblan L.W., Liu D.R. (2020). Genome editing with CRISPR–Cas nucleases, base editors, transposases and prime editors. Nat. Biotechnol..

[5] Gaj T., Gersbach C.A., Barbas C.F. (2013). ZFN, TALEN, and CRISPR/Cas-based methods for genome engineering. Trends Biotechnol..

[6] Ghosh D., Venkataramani P., Nandi S., Bhattacharjee S. (2019). CRISPR–Cas9 a boon or bane: The bumpy road ahead to cancer therapeutics. Cancer Cell Int..

[7] Kosicki M., Tomberg K., Bradley A. (2018). Repair of double-strand breaks induced by CRISPR–Cas9 leads to large deletions and complex rearrangements. Nat. Biotechnol..

[8] Zheng X., Li S.-Y., Zhao G.-P., Wang J. (2017). An efficient system for deletion of large DNA fragments in *Escherichia coli* via introduction of both Cas9 and the non-homologous end joining system from *Mycobacterium smegmatis*. Biochem. Biophys. Res. Commun..

[9] Ledford H. (2024). No CRISPR: Oddball ‘jumping gene’ enzyme edits genomes without breaking DNA. Nature.

[10] Yarnall M.T.N., Ioannidi E.I., Schmitt-Ulms C., Krajeski R.N., Lim J., Villiger L., Zhou W., Jiang K., Garushyants S.K., Roberts N. (2023). Drag-and-drop genome insertion of large sequences without double-strand DNA cleavage using CRISPR-directed integrases. Nat. Biotechnol..

[11] Rybarski J.R., Hu K., Hill A.M., Wilke C.O., Finkelstein I.J. (2021). Metagenomic discovery of CRISPR-associated transposons. Proc. Natl. Acad. Sci. USA.

[12] Durrant M.G., Perry N.T., Pai J.J., Jangid A.R., Athukoralage J.S., Hiraizumi M., McSpedon J.P., Pawluk A., Nishimasu H., Konermann S. (2024). Bridge RNAs direct programmable recombination of target and donor DNA. Nature.

[13] Siddiquee R., Pong C.H., Hall R.M., Ataide S.F. (2024). A programmable seekRNA guides target selection by IS1111 and IS110 type insertion sequences. Nat. Commun..

[14] Perry N.T., Bartie L.J., Katrekar D., Gonzalez G.A., Durrant M.G., Pai J.J., Fanton A., Martins J.Q., Hiraizumi M., Ricci-Tam C. (2025). Megabase-scale human genome rearrangement with programmable bridge recombinases. Science.

[15] Pelea O., Tálas A., Fernández Carrera J., Mathis N., van de Venn L., Yeh C.D., Kulcsár P.I., Marquart K.F., Weber Y., Gerecke S.E. (2026). Programmable genome editing in human cells using RNA-guided bridge recombinases. Science.

[16] Guo J., Song Z., Wang W., Huang S., Zhang Y., Li G., Huang X., Wang X., Liu J., Wu L. (2026). Optimization of IS621 recombinase/bridge RNA-directed recombination for precise insertion of large DNA fragments in human cells. Nat. Commun..

[17] Liu J., Aliaga Goltsman D.S., Alexander L.M., Khayi K.K., Hong J.H., Dunham D.T., Romano C.A., Temoche-Diaz M.M., Chadha S., Fregoso Ocampo R. (2025). Integration of therapeutic cargo into the human genome with programmable type V-K CAST. Nat. Commun..

[18] Witte I.P., Lampe G.D., Eitzinger S., Miller S.M., Berríos K.N., McElroy A.N., King R.T., Stringham O.G., Gelsinger D.R., Vo P.L.H. (2025). Programmable gene insertion in human cells with a laboratory-evolved CRISPR-associated transposase. Science.

[19] Liu B., Petti A., Zhou X., Cheng H., Gao J., Yee M., Qiao Y., Zhang Y., Zhou L., Wolfe S.A. (2026). Prime assembly with linear DNA donors enables large genomic insertions. Nature.

[20] Galiot L., Monger X.C., Vincent A.T. (2023). Studying the association between antibiotic resistance genes and insertion sequences in metagenomes: Challenges and pitfalls. Antibiotics.

[21] Siguier P., Gourbeyre E., Chandler M. (2014). Bacterial insertion sequences: Their genomic impact and diversity. FEMS Microbiol. Rev..

[22] Choi S., Ohta S., Ohtsubo E. (2003). A novel IS element, IS621, of the IS110/IS492 family transposes to a specific site in repetitive extragenic palindromic sequences in *Escherichia coli*. J. Bacteriol..

[23] Hiraizumi M., Perry N.T., Durrant M.G., Soma T., Nagahata N., Okazaki S., Athukoralage J.S., Isayama Y., Pai J.J., Pawluk A. (2024). Structural mechanism of bridge RNA-guided recombination. Nature.

[24] Na X.-M., Chen B.-T., Liu H., Lin Y.-T., Yuan Y., Chen T.-J., Xiong Y.-N., Zhang Y.-H., Yang N., Zhu F. (2026). ISPpu10 is a structure-gated bridge RNA recombinase that drives safe, large-scale genomic plasticity. bioRxiv.

[25] Patel J.R., Swartz S.E., Oromí-Bosch A., Yong L., LaTurner Z.W., Demaray J.E., Voelker A., Ono R., Vu P., Rao P. (2026). Bridge recombinase enables versatile rewriting of bacterial genomes. bioRxiv.

[26] Hiraizumi M., Athukoralage J.S., Perry N.T., Tsujimoto E., Shiojiri N., Nagahata N., Lee L., Sun G., Durrant M.G., Chandrasekaran S.S. (2026). Structural mechanism governing the directionality of bridge recombination. Nature.

[27] Durrant M.G., Fanton A., Tycko J., Hinks M., Chandrasekaran S.S., Perry N.T., Schaepe J., Du P.P., Lotfy P., Bassik M.C. (2023). Systematic discovery of recombinases for efficient integration of large DNA sequences into the human genome. Nat. Biotechnol..

[28] Fanton A., Bartie L.J., Martins J.Q., Tran V.Q., Goudy L., Kernick C., Durrant M.G., Wei J., Armour-Garb Z., Pawluk A. (2025). Site-specific DNA insertion into the human genome with engineered recombinases. Nat. Biotechnol..

[29] Fauser F., Arangundy-Franklin S., Davis J.E., Liu L., Schmidt N.J., Rodriguez L., Xia D.F., Nguyen N., Zhou Y., Scarlott N.A. (2026). Retargeted serine integrases for one-step, precise integration of large DNA sequences in human cells. Nat. Biotechnol..

[30] Tian X., Zhou B. (2021). Strategies for site-specific recombination with high efficiency and precise spatiotemporal resolution. J. Biol. Chem..

[31] Jinek M., Chylinski K., Fonfara I., Hauer M., Doudna J.A., Charpentier E. (2012). A programmable dual-RNA-guided DNA endonuclease in adaptive bacterial immunity. Science.

[32] Yang Y., Xu J., Ge S., Lai L. (2021). CRISPR/Cas: Advances, limitations, and applications for precision cancer research. Front. Med..

[33] Komor A.C., Kim Y.B., Packer M.S., Zuris J.A., Liu D.R. (2016). Programmable editing of a target base in genomic DNA without double-stranded DNA cleavage. Nature.

[34] Gaudelli N.M., Komor A.C., Rees H.A., Packer M.S., Badran A.H., Bryson D.I., Liu D.R. (2017). Programmable base editing of A•T to G•C in genomic DNA without DNA cleavage. Nature.

[35] Anzalone A.V., Randolph P.B., Davis J.R., Sousa A.A., Koblan L.W., Levy J.M., Chen P.J., Wilson C., Newby G.A., Raguram A. (2019). Search-and-replace genome editing without double-strand breaks or donor DNA. Nature.

[36] Murray J.B., Harrison P.T., Scholefield J. (2025). Prime editing: Therapeutic advances and mechanistic insights. Gene Ther..

[37] Anzalone A.V., Gao X.D., Podracky C.J., Nelson A.T., Koblan L.W., Raguram A., Levy J.M., Mercer J.A.M., Liu D.R. (2022). Programmable deletion, replacement, integration and inversion of large DNA sequences with twin prime editing. Nat. Biotechnol..

[38] Strecker J., Ladha A., Gardner Z., Schmid-Burgk J.L., Makarova K.S., Koonin E.V., Zhang F. (2019). RNA-guided DNA insertion with CRISPR-associated transposases. Science.

[39] Gao Y., Ma Y., Yu K., Liu Y., Gu B., Tang H., Yan W., Yang S., Su J., Wang X. (2026). Efficient and precise programmable DNA knock-in without double-strand breaks. Nature.

[40] Jung H., Jeong B., Kim Y.-W., Jung C., Lee S., Uhm H., Kim H., Oh Y.E., Park Y., Lee Y. (2026). Precise genomic integration of large DNA fragments by donor-directed annealing using prime editing. Nat. Biotechnol..

[41] Zhang X., Van Treeck B., Horton C.A., McIntyre J.J.R., Palm S.M., Shumate J.L., Collins K. (2025). Harnessing eukaryotic retroelement proteins for transgene insertion into human safe-harbor loci. Nat. Biotechnol..

[42] Chen Y., Luo S., Hu Y., Mao B., Wang X., Lu Z., Shan Q., Zhang J., Wang S., Feng G. (2024). All-RNA-mediated targeted gene integration in mammalian cells with rationally engineered R2 retrotransposons. Cell.

[43] Fell C.W., Villiger L., Lim J., Hiraizumi M., Tagliaferri D., Yarnall M.T.N., Lee A., Jiang K., Kayabolen A., Krajeski R.N. (2025). Reprogramming site-specific retrotransposon activity to new DNA sites. Nature.

[44] Edmonds K.K., Wilkinson M.E., Strebinger D., Chen H., Lash B., Schaefer C.C., Zhu S., Liu D., Zilberzwige-Tal S., Ladha A. (2025). Structure and biochemistry-guided engineering of an all-RNA system for DNA insertion with R2 retrotransposons. Nat. Commun..

[45] Rezazade Bazaz M., Ghahramani Seno M.M., Dehghani H. (2022). Transposase-CRISPR mediated targeted integration (TransCRISTI) in the human genome. Sci. Rep..

[46] Sternberg N., Hamilton D. (1981). Bacteriophage P1 site-specific recombination: I. Recombination between loxP sites. J. Mol. Biol..

[47] Broach J.R., Guarascio V.R., Jayaram M. (1982). Recombination within the yeast plasmid 2μ circle is site-specific. Cell.

[48] Jelicic M., Schmitt L.T., Paszkowski-Rogacz M., Walder A., Schubert N., Hoersten J., Sürün D., Buchholz F. (2023). Discovery and characterization of novel Cre-type tyrosine site-specific recombinases for advanced genome engineering. Nucleic Acids Res..

[49] Zhang Q., Azarin S.M., Sarkar C.A. (2022). Model-guided engineering of DNA sequences with predictable site-specific recombination rates. Nat. Commun..

[50] Mukhametzyanova L., Schmitt L.T., Torres-Rivera J., Rojo-Romanos T., Lansing F., Paszkowski-Rogacz M., Hollak H., Brux M., Augsburg M., Schneider P.M. (2024). Activation of recombinases at specific DNA loci by zinc-finger domain insertions. Nat. Biotechnol..

[51] Cautereels C., Smets J., De Saeger J., Cool L., Zhu Y., Zimmermann A., Steensels J., Gorkovskiy A., Jacobs T.B., Verstrepen K.J. (2024). Orthogonal LoxPsym sites allow multiplexed site-specific recombination in prokaryotic and eukaryotic hosts. Nat. Commun..

[52] Hew B.E., Gupta S., Sato R., Waller D.F., Stoytchev I., Short J.E., Sharek L., Tran C.T., Badran A.H., Owens J.B. (2024). Directed evolution of hyperactive integrases for site specific insertion of transgenes. Nucleic Acids Res..

[53] Nomura W. (2018). Development of toolboxes for precision genome/epigenome editing and imaging of epigenetics. Chem. Rec..

[54] Zuo Z., Billings T., Walker M., Petkov P.M., Fordyce P.M., Stormo G.D. (2023). On the dependent recognition of some long zinc finger proteins. Nucleic Acids Res..

[55] Singh A.K., Ramalingam S., Rao D.N., Chandrasegaran S. (2021). Genome editing revolution in life sciences. Resonance.

[56] Zhu J., Ma W., Huang Z., Zhang Q., Xie X., Yang X., Sun H. (2019). The application of genome editing technology. Biotarget.

[57] Bennett E.P., Petersen B.L., Johansen I.E., Niu Y., Yang Z., Chamberlain C.A., Met Ö., Wandall H.H., Frödin M. (2020). INDEL detection, the ‘Achilles heel’ of precise genome editing: A survey of methods for accurate profiling of gene editing induced indels. Nucleic Acids Res..

[58] Cruz-Becerra G., Kadonaga J.T. (2020). Enhancement of homology-directed repair with chromatin donor templates in cells. eLife.

[59] Xu H.R., Li T., Guo Y., Li H.F., Wang L., Zhang Z.Y., Wang X. (2015). Efficient assembly and verification of ZFNs and TALENs for modifying porcine *ApoE* gene. bioRxiv.

[60] Zhang H.X., Zhang Y., Yin H. (2019). Genome editing with mRNA encoding ZFN, TALEN, and Cas9. Mol. Ther..

[61] Sakono M., Oya T., Aoki M. (2023). Development of a transcriptional activator-like effector protein to accurately discriminate single nucleotide difference. ChemBioChem.

[62] Shankar S., Sreekumar A., Prasad D., Das A.V., Pillai M.R. (2018). Genome editing of oncogenes with ZFNs and TALENs: Caveats in nuclease design. Cancer Cell Int..

[63] Paquet D., Kwart D., Chen A., Sproul A., Jacob S., Teo S., Olsen K.M., Gregg A., Noggle S., Tessier-Lavigne M. (2016). Efficient introduction of specific homozygous and heterozygous mutations using CRISPR/Cas9. Nature.

[64] Zetsche B., Gootenberg J.S., Abudayyeh O.O., Slaymaker I.M., Makarova K.S., Essletzbichler P., Volz S.E., Joung J., van der Oost J., Regev A. (2015). Cpf1 is a single RNA-guided endonuclease of a class 2 CRISPR-Cas system. Cell.

[65] Abudayyeh O.O., Gootenberg J.S., Konermann S., Joung J., Slaymaker I.M., Cox D.B.T., Shmakov S., Makarova K.S., Semenova E., Minakhin L. (2016). C2c2 is a single-component programmable RNA-guided RNA-targeting CRISPR effector. Science.

[66] Abudayyeh O.O., Gootenberg J.S., Essletzbichler P., Han S., Joung J., Belanto J.J., Verdine V., Cox D.B.T., Kellner M.J., Regev A. (2017). RNA targeting with CRISPR–Cas13. Nature.

[67] Ai Y., Liang D., Wilusz J.E. (2022). CRISPR/Cas13 effectors have differing extents of off-target effects that limit their utility in eukaryotic cells. Nucleic Acids Res..

[68] Hu J.H., Miller S.M., Geurts M.H., Tang W., Chen L., Sun N., Zeina C.M., Gao X., Rees H.A., Lin Z. (2018). Evolved Cas9 variants with broad PAM compatibility and high DNA specificity. Nature.

[69] Landrum M.J., Lee J.M., Benson M., Brown G., Chao C., Chitipiralla S., Gu B., Hart J., Hoffman D., Hoover J. (2016). ClinVar: Public archive of interpretations of clinically relevant variants. Nucleic Acids Res..

[70] McCann J.L., Salamango D.J., Law E.K., Brown W.L., Harris R.S. (2020). MagnEdit-interacting factors that recruit DNA-editing enzymes to single base targets. Life Sci. Alliance.

[71] Wang Y., Zhou L., Liu N., Yao S. (2019). BE-PIGS: A base-editing tool with deaminases inlaid into Cas9 PI domain significantly expanded the editing scope. Signal Transduct. Target. Ther..

[72] Ellis K., Terry S.F. (2015). Dangerous liaisons: Connecting CRISPR/Cas9 to clinical science. Genet. Test. Mol. Biomark..

[73] Cwik B. (2017). Designing ethical trials of germline gene editing. N. Engl. J. Med..

[74] Rothschild J. (2020). Ethical considerations of gene editing and genetic selection. J. Gen. Fam. Med..

[75] Parums D.V. (2024). Editorial: Genome editing goes beyond CRISPR with the emergence of ‘Bridge’ RNA editing. Med. Sci. Monit..

[76] Ali Agha A.S.A., Al-Samydai A., Aburjai T. (2025). New frontiers in CRISPR: Addressing antimicrobial resistance with Cas9, Cas12, Cas13, and Cas14. Heliyon.

[77] Munsamy G., Ayres G., Greco C., Kam K., Minto-Cowcher G., St. John J., Bohnuud T., Bakalar M., Chow W., Pecoraro R. (2026). Designing AI-programmable therapeutics with the EDEN family of foundation models. bioRxiv.

